# Urine klotho is a potential early biomarker for acute kidney injury and associated with poor renal outcome after cardiac surgery

**DOI:** 10.1186/s12882-019-1460-5

**Published:** 2019-07-17

**Authors:** Yingying Qian, Lin Che, Yucheng Yan, Renhua Lu, Mingli Zhu, Song Xue, Zhaohui Ni, Leyi Gu

**Affiliations:** 1 Department of Nephrology, Renji Hospital, School of Medicine, Shanghai Jiao Tong University, 160 Pujian Road, Shanghai, 200127 China; 2 Department of Nephrology, Affiliated Hangzhou First People’s Hospital, Zhejiang University School of Medicine, Hangzhou, China; 3 Department of Nephrology, The Affiliated Hospital of Qingdao University, Qingdao, China; 4 Department of Cardiovascular Surgery, Renji Hospital, School of Medicine, Shanghai Jiao Tong University, Shanghai, China

**Keywords:** Acute kidney injury, Cardiac surgery, Klotho, NGAL, Early biomarker

## Abstract

**Background:**

Current paradigms of detecting acute kidney injury (AKI) are insensitive and non-specific. Klotho is a pleiotropic protein that is predominantly expressed in renal tubules. In this study, we evaluated the diagnostic and prognostic roles of urine Klotho for AKI following cardiac surgery.

**Methods:**

We conducted a prospective study involving 91 patients undergoing cardiac surgery. AKI was defined according to the AKIN definition. The renal outcomes within 7 days after operation were evaluated. Perioperative levels of urine Klotho and urine neutrophil gelatinase-associated lipocalin (NGAL) were measured by using ELISA.

**Results:**

Of 91 participants, 33 patients (36.26%) developed AKI. Of these AKI patients, 21 (63.64%), 8 (24.24%), and 4 (12.12%) were staged 1, 2, and 3, respectively. Serum creatinine in AKI patients began to slightly increase at first postoperative time and reached the AKI diagnostic value 1 day after operation.

Postoperative urine Klotho peaked at the first postoperative time (0 h after admission to the intensive care unit (ICU)) in patients with AKI, and was higher than that in non-AKI patients up to day 3. The AUC of detecting AKI for urine Klotho was higher than urine NGAL at the first postoperative time and 4 h after admission to the ICU. In a multivariate model, increased first postoperative urine Klotho may be an independent predictor for AKI occurrence following cardiac surgery. The concentrations of first postoperative urine Klotho were higher in AKI stage 2 and 3 than those in stage 1 (*p* < 0.05), and were higher in patients with incomplete recovery of renal function than those with complete recovery (*p* < 0.05).

**Conclusions:**

Urine Klotho may serve as an early biomarker for AKI and subsequent poor short-term renal outcome in patients undergoing cardiac surgery.

## Background

Acute kidney injury (AKI) remains a highly prevalent and serious complication of cardiac surgery. AKI develops in up to 40% of patients who undergo cardiac surgery [1, 2]. Despite decades of research, the mortality of cardiac surgery-associated AKI (CSA-AKI) remains high, even for those patients whose renal function has completely recovered [3]. A major reason for the disappointing outcome is the scarcity of early biomarkers. Current diagnosis of AKI is made based on the changes of serum creatinine (SCr) and urine output, which lack sensitivity and reliability [4]. First, SCr reflects the loss of glomerular filtration function rather than the renal tubular lesions. Second, SCr is vulnerable to several nonrenal factors such as sex, muscle mass, diet and hemodynamic alterations.

Klotho is a multifunctional protein that includes transmembrane and soluble forms [5]. The latter is almost entirely derived from membrane-bound Klotho shedding and has been found in serum, cerebrospinal fluid and urine [6–9]. Soluble Klotho functions as a hormone and plays a role of anti-oxidative stress [10], anti-apoptosis [11], and anti-fibrosis [12]. The renal tubular epithelial cells are the principal cells that contribute to Klotho synthesis and excretion [13, 14]. Klotho regulates transporters and ion channels through autocrine or paracrine to the urinary luminal side [9, 14]. Klotho in the urine can be derived from plasma and interstitium through transcytosis, and can also originate from the tubule through secretion rather than filtered across the glomerular barrier [9, 14].

To date, few studies have focused on the urine Klotho in prediction of AKI. In the present study, we firstly assessed the diagnostic and prognostic performance of urine Klotho in patients undergoing cardiac surgery.

## Methods

### Patients and samples

Patients who underwent cardiac surgery at the Cardiology Division of the Renji Hospital, School of Medicine, Shanghai Jiaotong University between 1st October 2012 and 30th June 2013 were enrolled. Patients with chronic kidney disease were excluded [15]. Further exclusion criteria included thyroid disease, preoperative usage of high-dose corticosteroids, pre-existing urinary intact infection, and missing clinical data.

The urine specimens were collected at before surgery and at 0 h, 2 h, 4 h, 1 d, 3 d, and 7 d after admission to the ICU. The blood specimens were collected preoperatively, as well as at 0 h, 1 d, 2 d, 3 d, and 7 d after arrival to the ICU. The first postoperative samples were collected at 0 h after arrival to the ICU within 4 h after surgery. Samples were quickly collected (stayed less than 4 h at 4 °C) and centrifuged at 3000 rpm for 5 min. The supernatants were aliquoted and frozen at − 80 °C.

### Variable definitions

Data including preoperative characteristics, surgical details, and postoperative complications were collected. Diagnosis and staging of postoperative AKI were according to AKIN criteria [16]. AKI was defined as an increase in SCr of at least 50% or more than 0.3 mg/dl from baseline within 48 h. The short-term renal outcome of AKI patients was evaluated according to changes in renal function on day 7 after operation. Complete recovery was defined as reduction of serum creatinine from the peak to less than 0.3 mg/dl [17]. The eGFR was calculated using Chronic Kidney Disease Epidemiology Collaboration (CKD-EPI) equation [18].

### Biomarker assays

We measured urine Klotho using Klotho ELISA kits (Immuno-Biological Laboratories Co, Tokyo, Japan). We measured urine NGAL using NGAL ELISA kits (R&D Systems, Inc., Minneapolis, MN, USA). Samples were used with one freeze/thaw cycle and were detected by a technician with a blind method. The intra- and inter-assay coefficient of variation for Klotho were both< 15% and for NGAL were both< 10%. The results were corrected for urine creatinine excretion.

### Statistical analyses

Continuous variables were expressed as mean ± standard deviation, and were compared using *t* test or one-way ANOVA followed by Tukey multiple comparison tests when they were normally distributed. Non-normally distributed continuous data were expressed as medians with interquartile range, and were analyzed using Wilcoxon rank sum test. Categorical variables were analyzed with Pearson χ^2^ test or Fisher’s exact test. We plotted the receiver operating characteristic (ROC) curve to measure the sensitivity and specificity of urine Klotho and urine NGAL for AKI prediction at different time cutoffs. The area under the ROC (AUC) was calculated to assess the ability of each biomarker to discriminate between patients developing and those not developing AKI after cardiac surgery. We conducted a univariate analysis for the predictors of AKI. Then, those variables with *p* < 0.05 were candidates for multivariate logistic regression models, including operation time and the first postoperative urine Klotho. In the multivariate analysis, we also adjusted for important covariates that predict AKI in the cardiac surgery setting [19, 20], including baseline SCr.

SPSS version 19.0 (SPSS Inc., Chicago, Ill., USA) and MedCalc version 18.5 (Ostend, Belgium) for windows software were used for analyses. *P* < 0.05 was considered to be statistically significant.

## Results

### Patient characteristics

A total of 103 patients were screened and 91 patients were included in the study. 12 patients were excluded for pre-existing urinary intact infection (*n* = 2), pre-existing CKD (*n* = 3), and missing clinical data (*n* = 7). The mean preoperative estimated glomerular filtration rate (eGFR) was 86.89 ± 15.55 ml/min/1.73m^2^. The average age was 61.79 ± 9.37 years. 33 patients (36.26%) developed AKI at a median time of 24 h post-surgery. 21 (63.64%), 8 (24.24%) and 4 (12.12%) AKI patients were staged into 1, 2 and 3, respectively. No patient required acute dialysis and 2 died before discharge. The baseline characteristics were showed in Table 1. No difference between AKI and non-AKI individuals was found in demographics and the types of operation. Patients who developed AKI had longer operation times than those who did not (5.5 (4.5, 6.25) h vs. 4.5 (4.00, 5.50) h, *p* < 0.05).

**Table 1:** Baseline characteristics of studied population undergoing cardiac surgery

	All (*n* = 91)	AKI (*n* = 33)	Non-AKI (*n* = 58)	*p* value
Preoperative condition				
Male, *n* (%)	58 (63.7%)	23 (69.7%)	35 (63.7%)	0.372
Age (year)^a^	61.79 ± 9.37	64.15 ± 9.37	60.45 ± 9.18	0.070
Weight (kg)^a^	62.11 ± 10.29	63.36 ± 10.06	61.38 ± 10.45	0.384
Hypertension, *n* (%)	33 (36.3%)	13 (39.4%)	20 (34.5%)	0.639
Diabetes, *n* (%)	14 (15.4%)	6 (18.2%)	8 (13.8%)	0.577
Hyperuricemia, *n* (%)	15 (16.5%)	6 (18.2%)	9 (15.5%)	0.742
Cerebrovascular disease, *n* (%)	4 (4.4%)	0 (0.0%)	4 (6.9%)	0.312
Hyperlipidemia, *n* (%)	6 (6.6%)	1 (3.0%)	5 (8.6%)	0.553
Peripheral vascular disease, *n* (%)	3 (3.3%)	2 (6.1%)	1 (1.7%)	0.615
Congestive heart failure, *n* (%)	13 (14.3%)	7 (21.2%)	6 (10.3%)	0.266
Contrast medium, *n* (%)	14 (15.4%)	7 (21.2%)	7 (12.1%)	0.245
Use of ACE inhibitors/ARBs, *n* (%)	25 (27.5%)	9 (27.3%)	16 (27.6%)	0.974
Hemoglobin (g/L)^b^	129.00 (116.00, 141.00)	124.00 (113.00, 141.00)	133.00 (120.00, 141.50)	0.219
Scr (umol/L)^a^	73.77 ± 13.88	76.09 ± 15.60	72.44 ± 12.76	0.230
eGFR (ml/min/1.73m^2^)^a^	86.89 ± 15.55	88.38 ± 14.35	84.27 ± 17.37	0.228
Intraoperative condition				
Type of surgery				
CABG, *n* (%)	32 (35.2%)	10 (30.3%)	22 (37.9%)	0.464
Single valve, *n* (%)	20 (22.0%)	6 (18.2%)	14 (24.1%)	0.509
Double valve, *n* (%)	19 (20.0%)	9 (27.3%)	10 (17.2%)	0.258
CABG plus valve surgery, n (%)	10 (11.0%)	4 (12.1%)	6 (10.3%)	1.000
CHD, *n* (%)	5 (5.5%)	2 (6.1%)	3 (5.2%)	1.000
Aortic aneurysm surgery, *n* (%)	5 (5.5%)	2 (6.1%)	3 (5.2%)	1.000
CPB, *n* (%)	67 (73.6%)	27 (81.8%)	40 (70.2%)	0.222
Operation time, h^b^*	5.00 (4.00, 6.00)	5.50 (4.50, 6.25)	4.50 (4.00, 5.50)	0.002
CPB time (min)^b^	84.00 (0.00, 115.00)	94.00 (56.00, 134.00)	76.50 (0.00, 112.25)	0.223
AXC time (min)^b^	46.00 (0.00, 69.00)	55.00 (11.50, 73.50)	46.00 (0.00, 66.50)	0.537
Cardiac arrest time (min)^b^	48.00 (0.00, 74.00)	60.00 (13.00, 75.00)	46.50 (0.00, 70.00)	0.526
Transfusion > 400 ml, n (%)	10 (11.0%)	4 (12.1%)	6 (10.3%)	1.000

^a^Data were presented as means ± standard deviation (SD) and the difference were calculated using *t* test. ^b^Data were presented as medians (IOR, interquartile range) and were analyzed using non-parametric Mann-Whitney U test. Categorical variables were presented as number (percentage of the column total) and were analyzed using Pearson χ2 test. *p < 0.05

*ACE* Angiotensin-converting enzyme, *ARB* Angiotensin receptor blockers, *SCr* Serum creatinine, *eGFR* Estimated glomerular filtration rate, *CABG* Coronary artery bypass grafting, *CHD* Congenital heart disease, *CPB* Cardiopulmonary bypass, A*XC* Aortic cross clamp

### Perioperative biomarkers concentrations

**Fig. 1:**
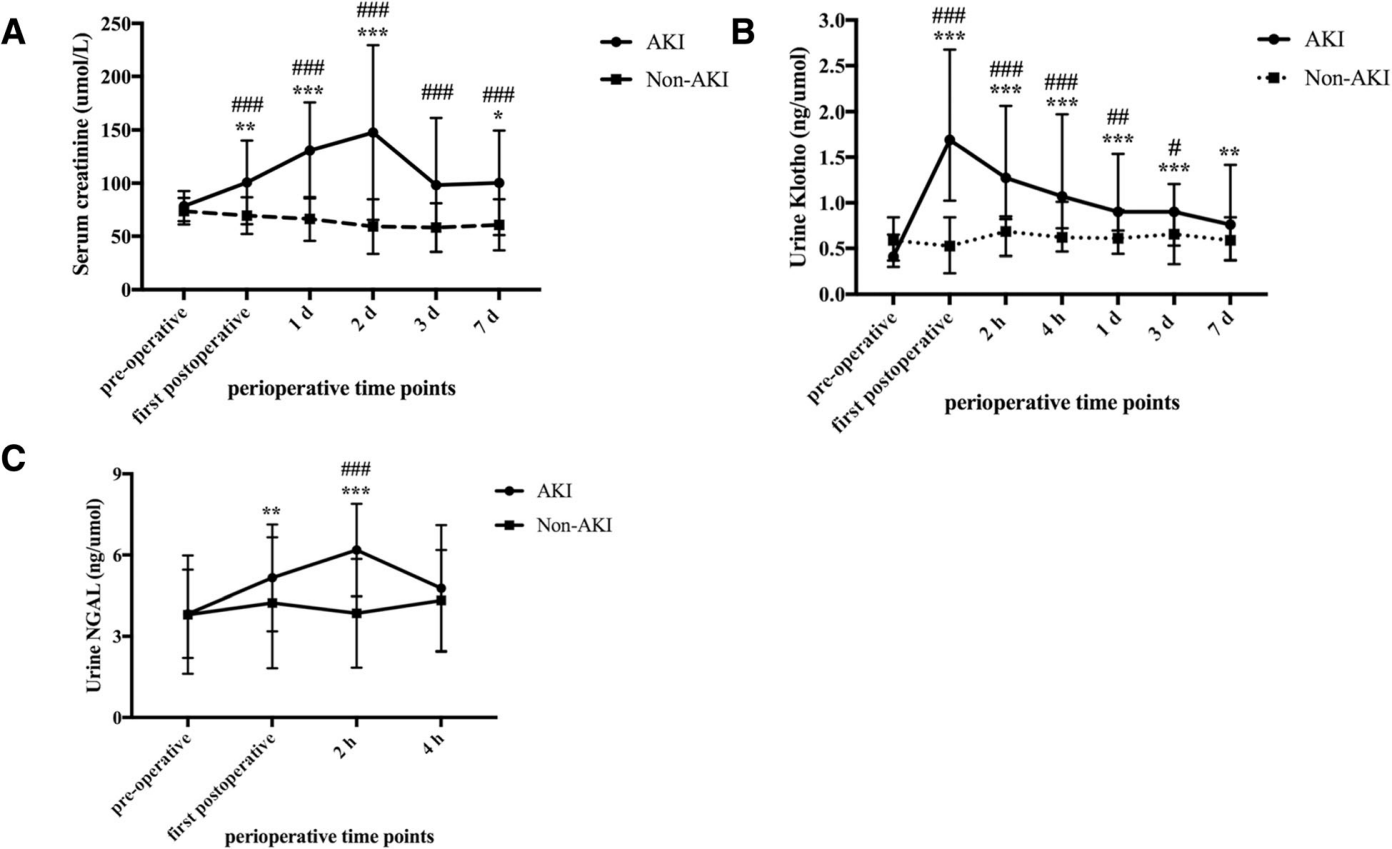
Perioperative levels of biomarkers in AKI and non-AKI patients. Concentrations of (**a**) serum creatinine, (**b**) urine Klotho, and (**c**) urine NGAL at indicated time points after cardiac surgery. The urine biomarkers were corrected for urine creatinine excretion. Data were presented as median with interquartile range. ^#^*P*-value < 0.05, ^##^*p*-value < 0.01, and ^###^*p*-value < 0.001 vs. non-AKI patients. ^*^P-value < 0.05, ^**^p-value < 0.01, and ^***^p-value < 0.001 vs. the preoperative levels

There was no difference in preoperative levels of Scr, urine Klotho (uKlotho) and urine NAGL (uNGAL) between patients with and without AKI. Serum creatinine in AKI patients began to slightly increase at first postoperative time (88.00 (78.35, 111.30) umol/L vs. preoperative: 76.80 (64.20, 90.45) umol/L, *p* < 0.01) and reached the AKI diagnostic value 1 day after arrival to the ICU (Fig. 1a). The levels of urine Klotho peaked at first postoperative time in patients with AKI and were 3 times higher than that before surgery (1.69 (1.02, 2.68) ng/umol vs. 0.41 (0.30, 0.65) ng/umol, *p* < 0.001, Fig. 1b). Further, urine Klotho levels in AKI patients were strikingly higher than those in non-AKI patients at the first postoperative time (1.69 (1.02, 2.68) ng/umol vs. 0.52 (0.23, 0.84) ng/umol, *p* < 0.01), and remained significantly higher up to day 3 postoperatively. The levels of urine NGAL in patients who developed AKI peaked at 2 h after admission to the ICU, but recovered at 4 h. Urine NGAL in patients who developed AKI was significantly higher than those who did not at 2 h (Fig. 1c).

### AUC analyses for cardiac surgery-associated AKI

We evaluated the AUCs for biomarkers in predicting AKI at indicated time points. As shown in Table 2, the AUC for uKlotho at the first postoperative time was 0.86 with the cutoff 0.86 ng/umol (sensitivity 93.9%, specificity 75.9%). Urine Klotho had higher AUCs than uNGAL at the first postoperative time and 4 h after arrival to the ICU (*p* < 0.001).

**Table 2:** AUC of biomarkers to predict AKI after cardiac surgery

	AUC (95% CI)	Cut-off (Sensitivity, Specificity)	*p* value
Urine Klotho (ng/umol)			
The first postoperative	0.86*** (0.78, 0.94)	0.86 (93.9, 75.9%)	< 0.001
2 h admission to the ICU	0.85 (0.76, 0.94)	0.82 (87.5, 77.6%)	< 0.001
4 h admission to the ICU	0.78*** (0.76, 0.94)	0.70 (90.6, 62.1%)	< 0.001
Urine NGAL (ng/umol)			
The first postoperative	0.59 (0.46, 0.71)	4.39 (72.7, 56.6%)	0.177
2 h admission to the ICU	0.78 (0.68, 0.88)	4.49 (87.9, 71.7%)	< 0.001
4 h admission to the ICU	0.57 (0.44, 0.70)	4.64 (57.6, 64.2%)	0.285

*AUC* Area under the ROC, *NGAL* Neutrophil gelatinase-associated lipocalin

***P < 0.001 compared with the AUCs of urine NGAL at indicated time points

### The first postoperative urine klotho was associated with the occurrence of AKI

As shown in Table 3, the first postoperative uKlotho was associated with AKI occurrence with unadjusted and adjusted odds ratio (OR) 3.36 (95% CI, 1.86 to 6.07) and 3.43 (95% CI, 1.87 to 6.28), respectively. The data also showed that the risk of AKI occurrence increased by 1.5 times for each additional hour of operation.

**Table 3:** Association of first postoperative urine Klotho with AKI

	AKI (33/91)	
	Unadjusted OR	Adjusted OR
Urine Klotho^a^ (per 1 ng/umol increase)	3.36 (1.86–6.07)***	3.43 (1.87–6.28)***
operation time^b^ (per 1 h increase)	1.58 (1.14–2.20)**	1.52 (1.03–2.24)*

***p < 0.001

Urine Klotho (continuous), operation time (continuous)

^a^Adjusted for operation time, baseline SCr

^b^Adjusted for baseline SCr and the first postoperative urine Klotho

### Perioperative levels of uKlotho among different stages of AKI

**Fig. 2:**
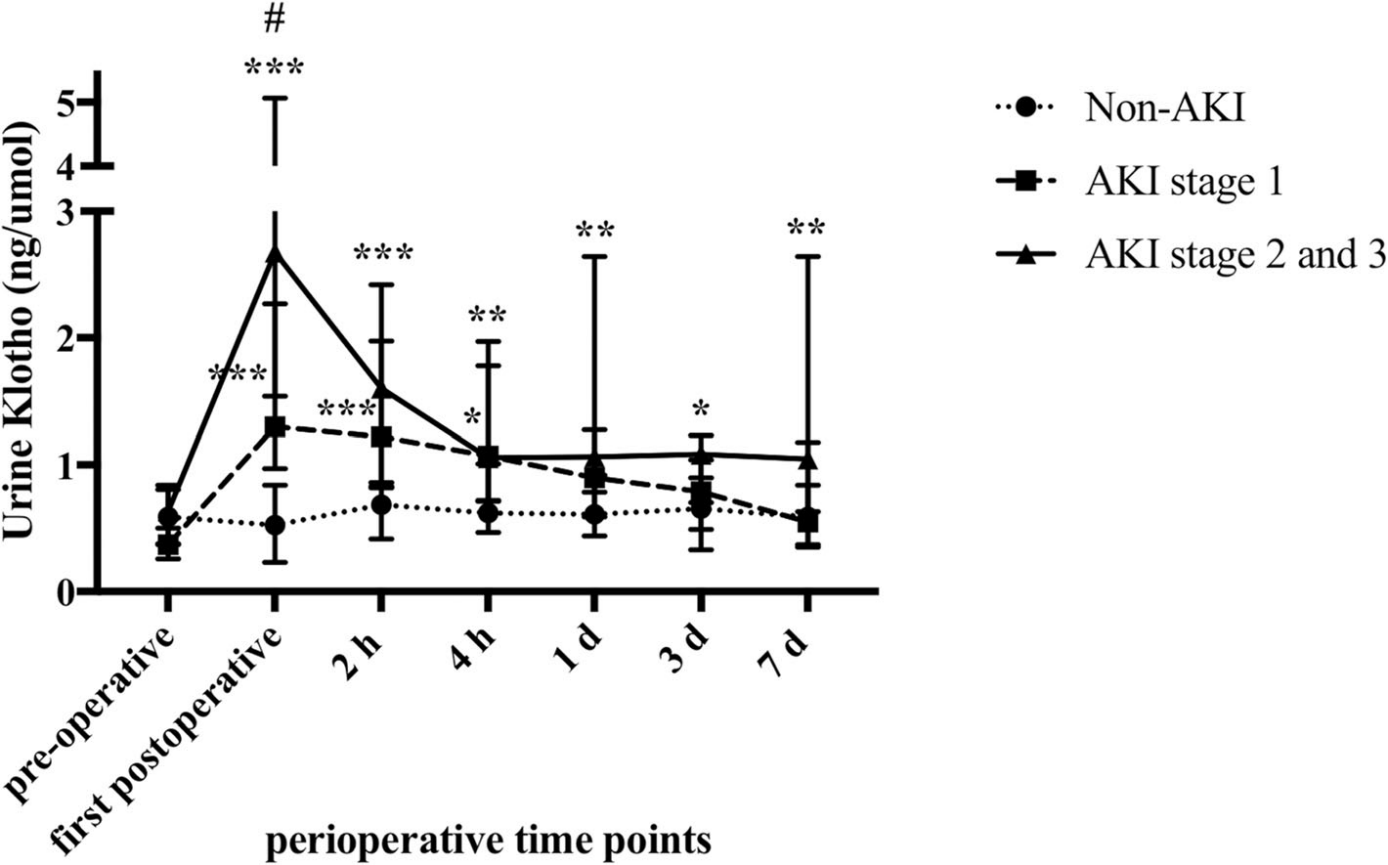
Perioperative levels of uKlotho among different stages of AKI. Patients were grouped as non-AKI, AKI stage 1, and AKI stage 2 and 3. The levels of uKlotho were analyzed. Urine Klotho was corrected for urine creatinine excretion. Data were presented as median with interquartile range. ^#^*P*-value < 0.05 vs. AKI stage 1 group. *P-value < 0.05, **p-value < 0.01, and ***p-value < 0.001 vs. non-AKI patients

Compared with non-AKI patients, patients with AKI stage 1 had significantly higher levels of uKlotho at the first postoperative time (Fig. 2). Patients who experienced AKI stage 2 and 3 had higher levels of first postoperative uKlotho than patients with stage 1 (2.68 (1.54, 5.06) ng/umol vs. 1.30 (0.97, 2.27) ng/umol, *P* < 0.05). Levels of uKlotho in patients with AKI stage 2 and 3 were markedly higher than that in non-AKI patients at the first postoperative time and remained higher up to day 7 (*P* < 0.01).

### Perioperative levels of uKlotho among non-AKI, AKI with complete recovery, and AKI with incomplete recovery

**Fig. 3:**
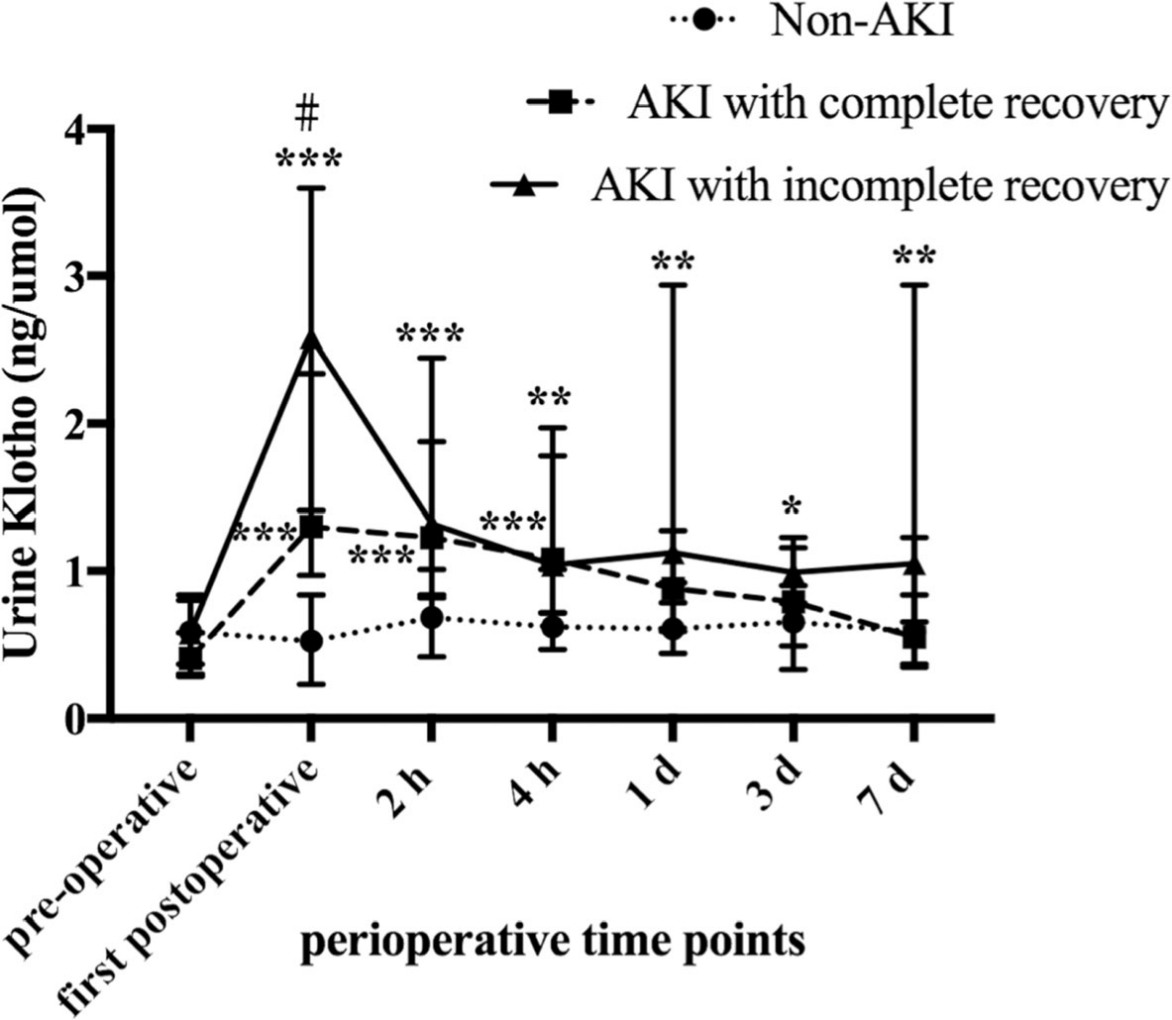
Perioperative levels of uKlotho among non-AKI, AKI with complete recovery, and AKI with incomplete recovery. Levels of uKlotho among 3 groups were analyzed. Data were presented as median with interquartile range. ^#^*P*-value < 0.05 vs. AKI with complete recovery. *P-value < 0.05, **p-value < 0.01, and ***p-value < 0.001 vs. non-AKI patients

As shown in Fig. 3, 21 (63.63%) AKI patients had complete recovery of renal function on day 7. The levels of first postoperative uKlotho were 0.52 (0.23, 0.84) ng/umol, 1.3 (0.97, 2.34) ng/umol, and 2.58 (1.41, 3.60) ng/umol in patients with non-AKI, complete recovery AKI, and incomplete recovery AKI, respectively (*p* < 0.01).

## Discussion

To our knowledge, we are the first to examine the time course of uKlotho among adults undergoing cardiac surgery. We found that the elevated first postoperative uKlotho may be an early predicator for the occurrence of CSA-AKI. Further, urine Klotho in patients with AKI stage 2 and 3 was higher than that in patients with AKI stage 1 at the first postoperative time. Urine Klotho in AKI patients with incomplete recovery of renal function was significantly higher than that in patients with complete recovery at the first postoperative time. 

AKI following cardiac surgery is a world health issue. Serum creatinine and urine output remains the gold standard for clinical diagnosis of AKI, although both were believed as unspecific markers for kidney injury. Novel biomarkers including NGAL, KIM-1 and interleukin-18 (IL-18) were evaluated for early diagnosis of AKI. Unlike Klotho that mainly expressed in kidney tubule, NGAL and IL-18 are nonspecific for kidney and are expressed in variety of tissues [4, 21]. Moreover, large, prospective, multicenter trails failed to show troponin-like diagnostic performance of plasma NGAL, urine NGAL, urine Kim-1, and urine IL-18 for AKI detecting with AUCs of less than 0.77 [22, 23]. Thus, efforts to validate potential markers are needed.

In animal experiments, Klotho deficiency in kidney tissues has been observed in both acute and chronic kidney injuries [24, 25]. More recently, a small study with 35 patients observed reduced serum Klotho in adults who developed AKI after cardiac surgery [26]. However, no significant difference of serum Klotho was found between patients with and without AKI on 1 day postoperatively and thereafter. Moreover, their study did not evaluate the change of urine Klotho and did not comment on the severity or renal outcome of AKI.

We firstly showed that uKlotho quickly increased as early as transferred to the ICU in patients who developed AKI later. Urine Klotho kept significantly higher in AKI patients than non-AKI patients until day 3 after surgery. The elevation of uKlotho occurred earlier than that of uNGAL demonstrated in the present cohorts and our previous data [27]. However, there was no difference in preoperative levels of uKlotho between patients with and without AKI. The early performance of urine Klotho may allow earlier detection of AKI and thus increase the success of therapeutic interventions.

To date, only two studies had examined uKlotho levels in patients or rodents with AKI. *Isidro* and his colleagues [28] examined the levels of uKlotho at 12 h post-cardiac surgery and showed a similar elevation of that in AKI patients (*n* = 15) compared with the healthy volunteers (*n* = 10). When comparing to the non-AKI (n = 15) group, the uKlotho levels in AKI patients increased but without significance. However, our results conflict with the reports from *Hu* et al. [24]. They found that urine Klotho levels in 17 AKI patients were significantly lower than that in 14 healthy controls by using immunoblotting assay. There are several reasons for the conflicted results. First, Hu did not describe the methods of pretreatment and the collecting time of urine samples. Klotho is unstable in urine and any additional freeze-thaw cycle decreases Klotho concentrations [29]. In the present study, urine samples were frozen at − 80 °C and were thawed for the first time for measurement of Klotho. Furthermore, uKlotho in patients with AKI was comparable with that in patients without AKI on 7 days post-surgery. Thus, a delayed collecting time may lead to different results. Second, the different causes of AKI may also lead to the contrary results. In the study of *Hu* et al., the causes of AKI are heterogeneous including sepsis, hypertension, CKD, nephrontoxin, pre-renal and others. Different measuring assays and fewer patients may also contribute the difference. Further researches with large sample size, multi-center and extensive time course are needed to clarify the change pattern of uKlotho.

Histologically, proximal tubular epithelial cells lose their brush border membrane as well as the Klotho protein expressed in the apical brush border, which may result in an acute increase of uKlotho at early AKI. With the progression of AKI, renal tubular epithelial cells were necrotic and exfoliated, accompanied with shedding of Klotho protein. This may contribute to the continuous elevation of uKlotho during AKI. We have previously showed similar phenomenon in the mouse model of AKI induced by renal ischemic-reperfusion injury [30]. Under these circumstances, the increase of uKlotho may indicate the severity of tubule injury. In support of this, we found that uKlotho was strongly associated with the occurrence of CSA-AKI and was significantly higher in AKI stage 2 and 3 than in stage 1.

Our study has several limitations. First, The present study is a single center study with a relatively small number of patients and short follow-up. Second, we did not compare the performance of predicting AKI between Klotho and other biomarkers besides NGAL. Third, the time points of uNGAL detection are insufficient. Additionally, the use of creatinine as a reference standard for biomarker assessment is imperfect for the known insensitivity and non-specificity.

## Conclusions

In conclusion, our study implies that first postoperative uKlotho may be an early predictor for the occurrence of AKI among patients undergoing cardiac surgery. The first postoperative uKlotho may be associated with the severity and prognosis of AKI. This may help to early identify at-risk patients before the progression to overt kidney injury and implement early interventions.

## Data Availability

The datasets used and/or analyzed during this study are available from the corresponding author on reasonable request.

## Abbreviations

ACE: Angiotensin-converting enzyme
AKI: Acute kidney injury
AKIN: Acute Kidney Injury Network
ARB: Angiotensin receptor blockers
AUC: Area under the ROC
AXC: Aortic cross clamp
CABG: Coronary artery bypass grafting
CHD: Congenital heart disease
CKD: Chronic kidney disease
CKD-EPI: Chronic Kidney Disease Epidemiology Collaboration
CPB: Cardiopulmonary bypass
CSA-AKI: Cardiac surgery-associated acute kidney injury
eGFR: Estimated glomerular filtration rate;
ICU: Intensive care unit
K/DOQI: Kidney Disease Outcome Quality Initiative
Kim-1: Kidney injury molecule-1
NGAL: Neutrophil gelatinase-associated lipocalin
OR: Odds rate
ROC: Receiver operating characteristic
SCr: Serum creatinine
UUO: Unilateral ureteral obstruction
